# BAU-Insectv2: An agricultural plant insect dataset for deep learning and biomedical image analysis

**DOI:** 10.1016/j.dib.2024.110083

**Published:** 2024-01-23

**Authors:** Imrus Salehin, Mahbubur Rahman Khan, Ummya Habiba, Nazmul Huda Badhon, Nazmun Nessa Moon

**Affiliations:** a Department of Computer Engineering, Dongseo University, 47 Jurye-ro, Sasang-gu, Busan, 47011, Republic of Korea; b Department of Computer Science and Engineering, Daffodil International University, Dhaka, 1216, Bangladesh; c Department of Food Processing and Preservation, Hajee Mohammad Danesh Science & Technology University, Dinajpur, 5200, Bangladesh; d Department of Industry 4.0 Convergence Bionics Engineering, Pukyong National University, 45 Yongso-ro, Nam-gu, Busan, Republic of Korea; e Faculty of Agriculture, Bangladesh Agricultural University, 2202, Mymensingh, Bangladesh

**Keywords:** Insect dataset, AI, Plant, Detection, Pest

## Abstract

“BAU-Insectv2” represents a novel agricultural dataset tailored for deep learning applications and biomedical image analysis focused on plant-insect interactions. This dataset encompasses a diverse collection of high-resolution images capturing intricate details of plant-insect interactions across various agricultural settings. Leveraging deep learning methodologies, this study aims to employ convolutional neural networks (CNN) and advanced image analysis techniques for precise insect detection, classification, and understanding of insect-related patterns within agricultural ecosystems. We mainly focus on addressing insect-related issues in South Asian crop cultivation. The dataset's extensive scope and high-quality imagery provide a robust foundation for developing and validating models capable of accurately identifying and analyzing diverse plant insects. The dataset's utility extends to biomedical image analysis, fostering interdisciplinary research avenues across agriculture and biomedical sciences. This dataset holds significant promise for advancing research in agricultural pest management, ecosystem dynamics, and biomedical image analysis techniques.

Specifications Table

**Table:** 

Subject	Artificial Intelligence, Agriculture Engineering, Plant Science
Specific subject area	AI and Agriculture Experiment: Image Data Analysis for Enhancing Food Production and Ensuring Food Security
Data format	Raw, Filtered.
Type of data	Filtered measurement data is stored as .zip (jpg) files.
Data collection	We employed our BAU-Insectv2 (Bangladesh Agricultural University Image Dataset) collected manually to compile a dataset consisting of 09 classes and more than 2000 raw data samples of varying quality. This dataset was specifically designed to encompass a wide array of insect species relevant to our research objectives. Gathering precise and high-quality research data can be a challenging task. For this study, we utilized two different image-capturing devices: a SAMSUNG smartphone camera and a Canon digital camera. The dataset includes diverse images featuring various backgrounds, heights, angles, crowd levels, arrangements, and combinations. The goal of this research is to explore methodologies for increasing data availability to facilitate robust experimentation and analysis in this domain.
Data source location	Faculty of Agriculture, Bangladesh Agricultural University, Mymensingh Bangladesh. Data collection Location: Local area of (Mymensingh, Dinajpur, Kushtia) Bangladesh
Data accessibility	Repository name: Mendeley data Data identification number: doi:10.17632/x2c6c8thdk.2 Direct URL to data: https://data.mendeley.com/datasets/x2c6c8thdk/2

## 1 Value of the Data


 

• BAU-Insectv2 image data serves as a scientific asset by enabling precise analysis of plant health, disease identification, and environmental impact on crops. This data aids in conducting comprehensive studies, developing predictive models, and advancing precision farming techniques for improved agricultural practices.
• High-quality agricultural images are invaluable for accurate plant disease identification, growth monitoring, and yield prediction. They enable precise analysis and aid in developing advanced AI models for improved crop management, disease prevention, and sustainable agricultural practices.
• In BAU-Insectv2 image attributes lie in their capacity to provide crucial information for agricultural analysis. Attributes like resolution, color depth, and spectral information allow for detailed examinations, aiding in the identification of diseases, growth patterns, and anomalies in crops. These attributes are essential for creating robust AI models and facilitating accurate predictions and decisions in agriculture.
• BAU-Insectv2 holds significant value in the realm of agricultural research due to its focus on South Asian crop pests. It offers a specific dataset, essential for understanding and addressing pest-related challenges unique to this region. Researchers can leverage this dataset to develop AI models that aid in pest identification, mitigation strategies, and predictive analysis, crucial for safeguarding crops and improving agricultural practices in South Asia.

## 2 Data Description

**Fig. 1:**
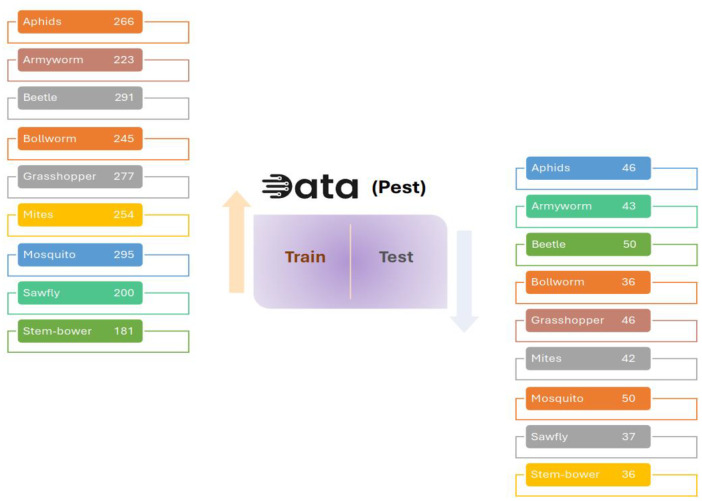
Data Class illustration with all instance amount.

The BAU-Insectv2 [1] Plant Insect Dataset compiles images depicting various insect species that impact plants and crops. Table 1 and Table 2 showcase the insect genera included in this dataset, along with the sample sizes for each genus. With categories representing distinct insect genera, the dataset comprises a range of images varying from 200 to 295 samples per genus (Fig. 1). This dataset serves as a beneficial tool for researchers and professionals engaging in insect classification, pest identification, and devising plant protection strategies. Its diverse insect species make the BAU-Insectv2 Plant Insect Dataset a valuable asset for studying and addressing agricultural challenges associated with insects, contributing significantly to ecosystem management. Targeting researchers and practitioners in entomology, agriculture, and computer vision, this dataset stands as an invaluable resource for advancing knowledge and practices in these domains.

**Table 1:** An outline of the BAU-Insectv2 dataset (Train File).

Genus	No. of samples
Aphids	266
Armyworm	223
Beetle	291
Bollworm	245
Grasshopper	277
Mites	254
Mosquito	295
Sawfly	200
Stem borer	181

**Table 2:** An outline of the BAU-Insectv2 Dataset (Test File).

Genus	No. of samples
Aphids	44
Armyworm	43
Beetle	50
Bollworm	36
Grasshopper	46
Mites	42
Mosquito	50
Sawfly	37
Stem borer	36

This dataset facilitates the creation and assessment of machine learning and deep learning models [2] aimed at insect classification [3], pest detection [4], and plant protection. The diverse sample sizes per genus offer a realistic and challenging environment for both training and testing models, mirroring the real-world distribution of insect species in agricultural contexts.

## 3 Experimental Design, Materials and Methods

**Fig. 2:**
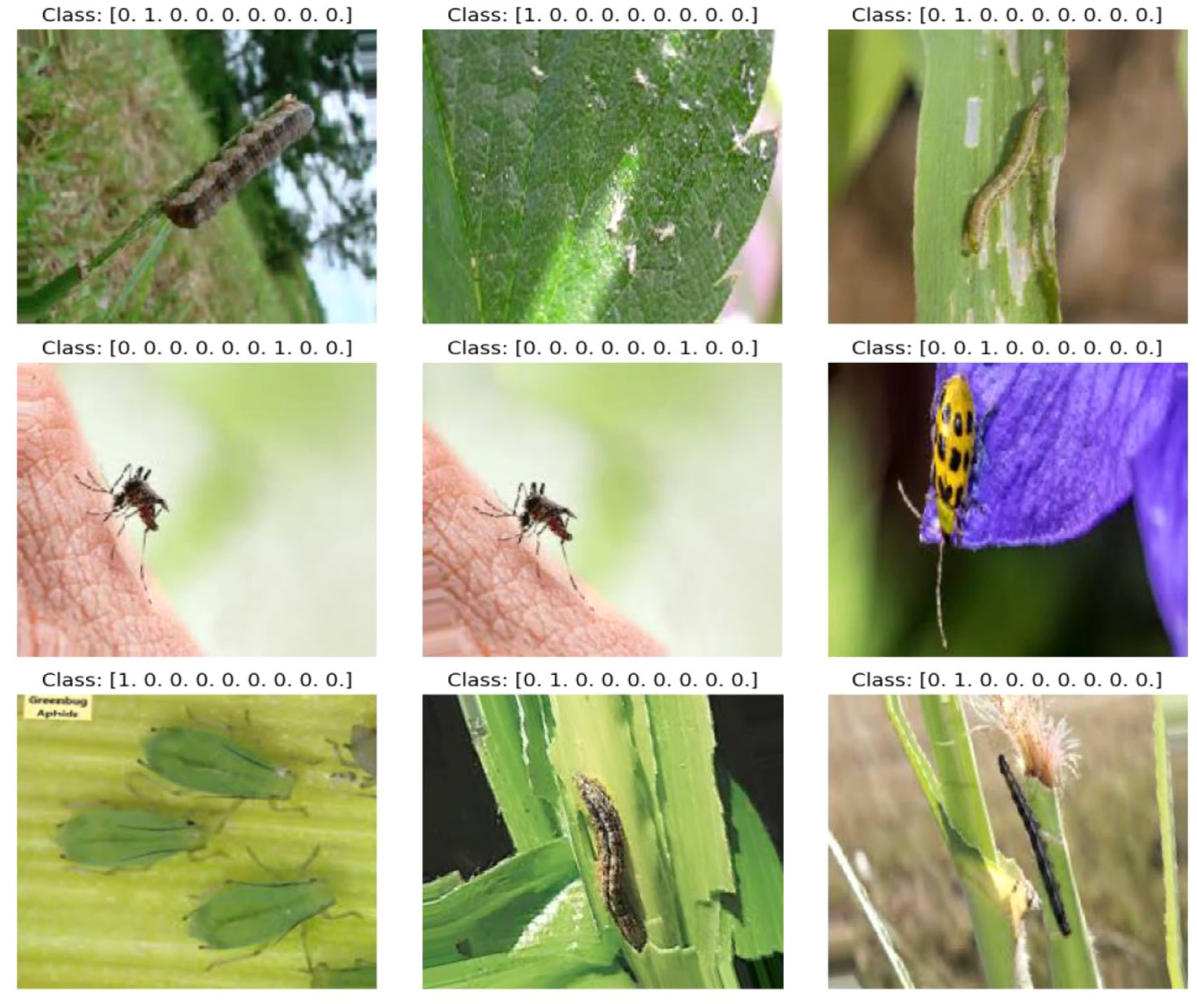
Class mapping with some random data from the BAU-Insectv2 dataset.

“BAU-Insectv2” denotes a scientific and regional version of a dataset and project concentrating on employing deep learning methodologies for analyzing images of plant insects. This initiative might involve utilizing advanced algorithms to process and comprehend visual data, aiming to enhance insect detection, classification, or other relevant aspects in the context of plant health [5] and ecosystem management [6]. Fig. 2 illustrates the results of the class mapping with some random data from the BAU-Insectv2 dataset.

### 3.1 Data visualization

In our pursuit of acquiring comprehensive insect image data within agricultural settings, we emphasize a systematic and scientific approach as foundational. Our process commences by setting clear research objectives that outline the target insect species, their habitats, and the expected environmental conditions. We then proceed with strategic site selection, considering various agricultural areas, crop types, and regional climates to encompass a diverse range of ecosystems.

### 3.2 Data processing

To collect insect images, we employ a methodical sampling strategy, utilizing precise data capture tools such as high-resolution cameras or smartphones equipped with quality lenses. The methodical sampling strategy for collecting insect images in detail is as follows:

The context of collecting insect images refers to a systematic and well-thought-out approach to selecting and capturing images of insects in their natural habitat. It involves several key steps:

a. Target Identification: This involves identifying specific insect species or categories that are the focus of the study. It could be certain types of insects or a broader range of species.
b. Site Selection: Choosing appropriate locations where the target insects are likely to be found. This involves considering diverse habitats, ecological niches, and environmental conditions.
c. Timing: Timing the image capture to coincide with the peak activity or prevalence of the identified insects. This could mean specific seasons, times of the day, or certain weather conditions when the insects are more active or visible.
d. Data Capture Tools: Using high-quality tools like high-resolution cameras or smartphones with quality lenses capable of capturing detailed images of insects. These tools help ensure that the images collected are clear, detailed, and suitable for analysis.
e. Image Capture Method: Employing consistent and meticulous methods for capturing images. This includes taking photographs from various angles, distances, and orientations to capture comprehensive and diverse data.
f. Consistency in Recording: Maintaining consistency in recording metadata associated with the images. This includes details such as location, date, time, environmental conditions, and any other relevant information that can aid in analysis.
g. Ethical Considerations: Adhering to ethical guidelines and regulations related to the collection of insects and their images. Respecting the environment, obtaining necessary permissions, and ensuring the well-being of the insects during the image capture process are crucial aspects.

During our field expeditions, which coincide with peak insect activity, we meticulously capture images from various angles to ensure thorough coverage. Consistency in image capture parameters and comprehensive metadata recording, encompassing location, date, time, and environmental variables, remains crucial for effective dataset management and subsequent analysis. Throughout our process, we maintain a commitment to upholding ethical guidelines and adhering to legal regulations governing insect collection and image capture. This commitment ensures scientific integrity and compliance with local regulations.

### 3.3 Possible machine learning and deep learning algorithms

For image data related to plant-insect analysis, several AI algorithms can be employed:

(a) Convolutional Neural Networks (CNN): CNNs excel in image recognition tasks by extracting hierarchical features from images. They consist of convolutional layers that detect patterns, followed by pooling layers to reduce dimensionality and fully connected layers for classification.
(b) Transfer Learning (TL): TL involves leveraging pre-trained models like VGG, ResNet, or Inception, and fine-tuning them on specific insect image datasets. This method speeds up training and enhances performance, especially with limited data.
(c) Recurrent Neural Networks (RNN): RNN process sequential data, useful for time-series or sequential insect behavior analysis. Long Short-Term Memory (LSTM) networks, a type of RNN, capture dependencies in sequential insect image data.
(d) Generative Adversarial Networks (GAN): GAN generates synthetic images that resemble real insect images. They consist of a generator creating images and a discriminator distinguishing between real and generated images, beneficial for data augmentation or creating diverse datasets.
(e) Attention Mechanisms: These mechanisms, like Transformer models, focus on specific parts of the image crucial for classification. They are effective for identifying intricate patterns in insect images.

Each of these algorithms offers unique advantages in processing and analyzing plant insect image data, catering to diverse requirements in insect recognition, behavior analysis, or dataset enhancement.

### 3.4 Future works

Future work in multi-domain applications [7] involving plant insect image analysis using deep learning could entail several facets:

(a) Cross-Domain Collaboration: Collaborating with experts from diverse fields like entomology, agriculture, or ecology to integrate domain-specific knowledge into deep learning models. This collaboration could enhance the models' understanding of insect behavior and ecology.
(b) Transferable Models: Developing transferable models that can adapt to different insect species or plant environments. These models would generalize across various domains, aiding in broader insect analysis applications.
(c) Multimodal Integration: Exploring the integration of multiple data modalities (such as visual, environmental, or sensor data) to enrich the analysis. Combining image data with other domain-specific information could yield comprehensive insights into insect-plant interactions.
(d) Robustness and Adaptability: Focusing on robust and adaptable models capable of handling variations in environmental conditions or insect populations. Adaptive models could account for diverse scenarios and evolving insect behaviors.
(e) Ethical and Environmental Implications: Researching the ethical implications of insect analysis and its impact on ecological systems. Ensuring that advancements in insect analysis contribute positively to ecosystem conservation and sustainable agriculture practices.

By embarking on multi-domain explorations, future research can broaden the scope of plant-insect image analysis, fostering collaborations across disciplines and addressing complex challenges in ecological conservation and agriculture.

In conclusion, the synergy between deep learning techniques and plant-insect image analysis promises a transformative trajectory, fostering interdisciplinary collaborations and ethical stewardship for a sustainable future in agriculture and ecosystem management.

## Limitations

The ``BAU-Insectv2” dataset, while a valuable resource for deep learning and biomedical image analysis, presents certain limitations that warrant consideration in research endeavors. Primarily, its limited diversity across geographic regions and insect species could restrict the models' applicability in varied agricultural settings. Potential inconsistencies or quality variations in annotations may impact the reliability of model predictions, particularly concerning complex insect behaviour or less-represented species. Ethical considerations, encompassing insect handling and environmental impact, demand careful attention to ensure responsible research conduct. Additionally, the dataset's computational demands and its relevance to biomedical analysis necessitate further validation and exploration.

## Ethics Statement

The authors have adhered to the ethical standards for Data in Brief publication. They affirm that their work did not entail data collection from human subjects, animal experiments, or social media platforms.

## Credit Author Statement

**Imrus Salehin:** Investigation, Software, Visualization, Writing - original draft, Writing - review & editing; **Mahbubur Rahman Khan**: Conceptualization, Methodology, Writing - review & editing; **Ummya Habiba and Nazmul Huda Badhon:** Data curation, Data cleaning, Data Filter, and Data Collection; and **Nazmun Nessa Moon:** Supervision and Review.

## Data Availability

BAU-Insectv2 Agricultural Plant Insect Dataset (Original data) (Mendeley Data)
